# Applicator-Based Versus Bulk-Bottle Delivery of the Same Alcoholic Chlorhexidine: A Single-Center, Non-Randomized Pilot Micro-Costing Study of Two Surgical Skin-Antisepsis Workflows

**DOI:** 10.3390/healthcare14203456

**Published:** 2026-10-15

**Authors:** Francisco Guillen-Grima, Maria Pilar Garcia-Garcia, Laura Guillen-Aguinaga, Belen Romanco-Moreno, Ander Eizaguirre-Artolozaga, Enrique Aguinaga-Ontoso

**Affiliations:** 1 Department of Preventive Medicine, Clínica Universidad de Navarra, 28027 Madrid, Spain; 2 Department of Preventive Medicine, Clínica Universidad de Navarra, 31008 Pamplona, Spain; 3 Healthcare Research Institute of Navarre (IdiSNA), 31008 Pamplona, Spain; 4 CIBER in Epidemiology and Public Health (CIBERESP), Institute of Health Carlos III, 28029 Madrid, Spain; 5 Department of Health Sciences, Public University of Navarra, 31008 Pamplona, Spain; 6 Department of Otolaryngology—Head and Neck Surgery, Clínica Universidad de Navarra, 28027 Madrid, Spain; 7 Operating Rooms Department, Clínica Universidad de Navarra, 31008 Pamplona, Spain; 8 Independent Researcher, 92110 Clichy, France; 9 Department of Sociosanitary Sciences, University of Murcia, 30120 Murcia, Spain; aguinaga@um.es; 10 Department of Preventive Medicine, Virgen de la Arrixaca University Clinical Hospital, 30120 Murcia, Spain

**Keywords:** surgical skin antisepsis, chlorhexidine-alcohol, single-use applicator, operating room workflow, micro-costing, waste reduction, perioperative resource use

## Abstract

**Highlights:**

**What are the main findings?**

- With the same alcoholic chlorhexidine, the bulk-bottle protocol required two handling steps (decanting onto a cloth and blotting excess with gauze, 39.8 s combined) that a pre-filled applicator eliminates by construction.
- Residual alcoholic chlorhexidine was 72.8% lower and total measured waste 27.9% lower with the applicator workflow; within the bulk-bottle arm, no difference in antiseptic consumption was detected between larger and smaller prepared fields in this small comparison.
- Among comparable procedures (unilateral hernia repair), slower execution of shared steps offset the structural saving: total workflow time differed by −7.3 s (95% CI −33.0 to +19.6).

**What are the implications of the main findings?**

- The material cost difference (€1.17–€3.26 per procedure) depends on reprocessing accounting and reverses if reprocessing is valued below about 78% of its average allocated cost; the larger €7.57 accounting difference is 85% time-dependent, realizable only if released minutes become usable activity, and did not persist among comparable procedures.
- A confirmatory study should cross operators over workflows, record prepared field size and delivered volume, and include drying time in the endpoint.

**Abstract:**

Background/Objectives: Delivery format may change the resources consumed by surgical skin antisepsis even when the antiseptic is unchanged. We compared two workflows using identical 2% chlorhexidine in 70% isopropyl alcohol: a single-use pre-filled applicator and our center’s bulk-bottle protocol. Methods: Single-center, non-randomized pilot micro-costing study (hospital perspective, €2024) of 20 elective abdominal procedures (10 per workflow) performed by non-overlapping surgeon groups. Time was measured stepwise to completion of application, excluding the air-drying interval; waste was weighed. Results: Two bulk-protocol steps, decanting onto a cloth and blotting excess with gauze (39.8 s combined), have no applicator counterpart. Residual alcoholic chlorhexidine was 72.8% lower and total waste 27.9% lower (−42.3 g; 95% CI −62.1 to −22.6). Materials differed by €1.17 per procedure (€3.26 on the same regulatory basis), a difference that reverses if reprocessing is valued below about 78% of its average allocated cost. Aggregate workflow time differed by −29.8 s (95% CI −61.4 to −1.9) and total cost, 85% of it time valued and realizable only if released minutes become usable activity, by −€7.57 (−14.38 to −1.56). Case mix biased both in favor of the applicator: among unilateral hernia repairs (seven vs. four) they fell to −7.3 s (−33.0 to +19.6) and −€2.74 (−8.26 to +3.05), whereas waste differences were unchanged. Operator nesting makes intervals for time, cost and liquid waste likely too narrow. Conclusions: With the same antiseptic, the applicator workflow removes two handling steps and was associated with less wasted antiseptic. Whether it saves time or money requires a study crossing operators over workflows and timing drying.

## 1. Introduction

Surgical site infections (SSIs) remain a major cause of postoperative morbidity and cost, with a pooled global prevalence of 2.5% and an estimated 14–16% share of all healthcare-associated infections [1]. Preoperative skin antisepsis with an alcohol-based preparation is a core preventive measure: the World Health Organization recommends alcohol-based chlorhexidine gluconate [2], CDC/HICPAC and SHEA/IDSA/APIC recommend an alcohol-containing agent [3,4], and NICE lists alcoholic chlorhexidine as the usual first choice [5].

Much of the evidence favoring chlorhexidine compares it in alcohol with aqueous povidone–iodine, so that vehicle and agent change together [6,7,8]. When both arms are alcohol-based, randomized trials have not shown a consistent advantage for chlorhexidine [9,10,11,12], and the SHEA/IDSA/APIC update states that the optimal antiseptic to combine with alcohol has not been established [4]. Alcoholic chlorhexidine is therefore a well-supported option rather than a uniformly superior one. In the present study it was the routine antiseptic, and the question was not which antiseptic to use but how to deliver it.

Once the agent has been chosen, the delivery format may still affect workflow, material use and waste. Bulk-bottle protocols require decanting and ancillary materials, some of which are reprocessed, whereas single-use applicators deliver a predefined volume [5,13,14], and NICE has identified the clinical and cost-effectiveness of different application modes as a research priority [5]. Direct comparisons of delivery formats have addressed microbiological or compliance outcomes. With the same chlorhexidine–alcohol formulation, applicator and gauze application reduced resident skin flora similarly in volunteers; the authors suggested advantages in standardization, time and resource use but did not measure them [15]. Other studies examined compliance with instructions, drying time or skin coverage [16,17,18], not procedure-level workflow, waste or cost.

Economic and environmental evidence is indirect. Because operating rooms are resource-intensive, small per-procedure differences can become substantial across a surgical caseload [19,20]. Published cost comparisons changed the antiseptic agent together with the delivery system [21] or focused on downstream infection costs [22], and a 2025 scoping review found no study measuring the cost or environmental impact of a branded applicator against generic chlorhexidine–alcohol [23]. Life-cycle assessments show that reusable devices are not always environmentally preferable [24,25], and the one analysis of applicator versus bulk-bottle preparation compared different antiseptic agents and modeled rather than observed resource use [26].

This prospective pilot micro-costing study therefore compared two complete skin-antisepsis workflows using the same alcoholic chlorhexidine, a pre-filled applicator and the study center’s bulk-bottle protocol, in terms of workflow time, waste and per-procedure cost. It evaluated operational and resource-use outcomes, not antimicrobial effectiveness or SSI incidence.

## 2. Materials and Methods

### 2.1. Organization of the Study and Piloting

This prospective, observational pilot study was conducted during routine elective surgery at a single center, without changes to surgical indication or clinical care. In a preliminary phase (February–March 2024), 50 general surgery procedures (25 per system) were observed to standardize the observation protocol, timing intervals, waste measurement and application technique. Surgical staff in both arms were then trained in the same 30 s back-and-forth technique at the abdominal site, followed by complete air drying, as specified by the Spanish Zero Surgical Infection (IQZ) protocol and the applicator’s instructions for use [27,28,29]. Standardization was intended to reduce variation in technique, not to imply superiority of back-and-forth friction.

The comparative phase included 20 elective abdominal general surgery procedures in adults (10 per workflow) in which 2% alcoholic chlorhexidine was indicated as part of routine care; procedures in patients with chlorhexidine allergy and urgent or emergency operations were excluded. Procedures were enrolled consecutively, as observer availability allowed, until 10 had been accrued in each arm. There was no randomization: surgeons used the protocol they routinely preferred. Eleven surgeons contributed, two per procedure, and none contributed to both arms, so operators were entirely nested within arms and operator and delivery-system effects cannot be separated; operator identity was not recorded on the observation sheets. Observation was sequential rather than concurrent. The bulk-bottle procedures were observed on 3, 9 and 10 April 2024 (four, one and five procedures) and the applicator procedures on 6, 7 and 8 May and 12 June 2024 (three, three, two and two), 26 days apart. This sequence followed the operating schedule and observer availability, not any change in practice, staffing or protocol; with twelve operating rooms and parallel general surgery lists, procedures observed on the same day were not necessarily performed in the same room or by the same team.

Both workflows began with a scrub with aqueous chlorhexidine gluconate (40 mg/mL) and removal of its excess with a reusable washcloth, followed by antisepsis with 2% alcoholic chlorhexidine. In the bulk-bottle protocol, the alcoholic solution was poured onto a reusable cloth for application, and excess was removed with sterile gauze; neither step exists with a pre-filled applicator. The transfer of solution and the removal of excess follow from the delivery format, whereas the particular ancillary items and their on-site reprocessing reflect local practice: a center using disposable items, or none, would retain the first kind of difference and lose much of the second. The comparison is therefore between two complete workflows as practiced at the study center, not between an applicator and every possible bulk-bottle implementation.

No formal sample size calculation was performed. The sample size was set by the number of procedures that could be observed in full without interfering with operating room activity. The study was designed to establish the feasibility of the measurements and to provide first estimates of workflow time, waste and cost, and of their variability, so that a confirmatory study can be adequately powered; the analyses are therefore estimation rather than hypothesis testing.

### 2.2. Measurement of Time

For each case, the duration of the predefined stages of the skin antisepsis procedure was measured prospectively using a stopwatch. The timed interval began with the start of material preparation for skin antisepsis and ended with the completion of antiseptic application. It did not include the subsequent air-drying interval, which the standardized protocol requires before draping and which was neither timed nor modeled. The endpoint reported throughout is therefore measured skin-antisepsis workflow time, and not the interval from the start of preparation to readiness for draping or incision. Because volume delivered to the skin was not an endpoint in either arm, differences in evaporation time cannot be excluded, and differences in measured workflow time should not be equated with differences in operating room time before incision.

### 2.3. Measurement of Residuals

After each procedure, all procedure-related residual material was collected and weighed on a calibrated electronic balance. Four variables were recorded per procedure: (i) residual alcoholic chlorhexidine, defined as antiseptic made available for the procedure but not delivered to the skin, obtained in both arms as a post-use weight less a dry reference weight and converted to volume at 0.88 g/mL; (ii) aqueous chlorhexidine discarded after the pre-scrub (mL); (iii) solid waste, the dry mass of discarded non-liquid items (packaging, the pre-scrub sponge and, in the bulk-bottle arm, the per-procedure share of the emptied bottle); and (iv) total measured waste, the gross weight of all discarded material, which also includes the mass retained by impregnated items and is therefore not the sum of the other components. In the bulk-bottle arm, the bottle was weighed before and after each procedure, and the gallipot and every absorbent item were weighed dry before use and again after use. In the applicator arm, residual antiseptic was the post-use mass of the device less its dry mass (41.12 g: the 64.00 g filled mass less the 26 mL nominal fill). The 26 mL applicator was the format stocked by the hospital and was used in all procedures; a 10.5 mL format was commercially available but not stocked at the center. Reusable items were reprocessed rather than discarded and are excluded from all waste endpoints. Item-level definitions and weighing procedures are given in Supplementary Section S1.

### 2.4. Economic Analysis

Costing approach. We followed the recommendations derived from systematic appraisals of micro-costing in health care [30,31] and in surgery [32]: resource use was identified and measured bottom-up at the level of the individual procedure by direct observation [33], resource quantities and unit costs are reported separately, and the perspective, price year and sensitivity analyses are stated explicitly. Micro-costing was preferred to gross costing because the differences between the two workflows lie in individual handling steps and consumables that aggregate hospital cost data would not resolve [34].

Perspective and time horizon. We conducted a micro-costing analysis from the hospital provider perspective on a per-procedure basis. The time horizon was limited to intraoperative skin antisepsis only. All costs are valued and reported in 2024 euros (€2024). No discount was applied, given the intraoperative, sub-year time horizon.

Basis of valuation. Personnel and operating room time were valued by applying institutional unit costs per minute to the observed workflow duration. These components are opportunity costs: shorter procedures do not by themselves reduce salaries or fixed operating room costs, and released minutes become a realizable saving only if they are aggregated and converted into additional activity. Material costs, by contrast, are avoidable per procedure. The two are therefore reported separately, and incremental costs are accounting costs under the stated valuation, not an immediate reduction in expenditure.

Cost components and unit costs. Material unit prices were taken from the Hospital Pharmacy price list current at the time of data collection, and personnel and operating room rates from the hospital’s Analytical Accounting and Profitability Department (Table 1). Personnel rates are fully loaded institutional costs rather than individual remuneration. Standard staffing during skin antisepsis comprised two surgeons, one anesthesiologist and three nurses (scrub, circulating and anesthesia), so that the personnel cost per minute was three times the medical rate plus three times the nursing rate. Because physician seniority was not recorded, the base case assumed senior medical staff, the valuation most favorable to the faster workflow; alternative staffing complements, a zero valuation of time and the tariff structure of a second hospital of the same institution were examined as scenarios (Supplementary Table S5).

Material cost calculation. Per-procedure material cost was the sum of quantity × unit price across items (Table 2). The bulk bottle was apportioned across three procedures, and reprocessing of reusable items was allocated by their tray-space share of a €45 sterilization container (Supplementary Section S2). In the applicator arm, material cost consisted solely of the single-use applicator (ChloraPrep^®^, Becton Dickinson, Franklin Lakes, NJ, USA). Unused solution contributed to waste but not to material cost. Total cost per procedure was the sum of material, personnel and operating room costs, and incremental cost was the mean difference between arms (applicator minus bulk bottle).

The bulk product used at our institution during the study period was registered as a biocide rather than a licensed medicinal product, which may affect its acquisition price relative to a medicinally authorized chlorhexidine–alcohol formulation and should be considered when transferring these unit costs to other settings. A sensitivity scenario costing both arms on the same regulatory basis is reported below.

Sensitivity and scenario analyses. Material prices, wage rates and the operating room rate were each varied by ±20% (one-way analysis); staffing complements and the second site’s tariffs were examined as scenarios; break-even values were calculated for each parameter, including the reprocessing allocation; and the applicator price and the combined time rate were varied jointly (two-way analysis). These analyses are reported in Supplementary Tables S4–S7 and Figures S1–S3. In a separate regulatory scenario, the bulk-bottle arm was re-costed with a medicinal-grade product of identical composition, because antiseptics for preoperative skin preparation have been reclassified in Spain as medicinal products for human use [35], consistent with European legislation [36,37]; biocide-registered stock was still in routine use during data collection. Because licensed products are for single-patient use, the full bottle price (€3.20, 100 mL) was attributed to one procedure (Table 2).

### 2.5. Statistical Analysis

Continuous variables are summarized as means and standard deviations. The principal estimand is the difference in arithmetic means between arms (applicator minus bulk bottle), because the consequence of adopting one workflow across a caseload is the number of procedures multiplied by the mean difference; the sensitivity analyses are built on the same means. Each difference is reported with bias-corrected and accelerated (BCa) and percentile bootstrap 95% confidence intervals from the same 10,000 resamples, stratified by delivery system (Mersenne Twister, seed 2,000,000). An independent implementation in Python (SciPy 1.17) reproduced the percentile intervals but not the BCa limits, whose acceleration constant is unstable with ten observations per arm; the percentile interval is therefore the more transportable of the two. Differences were also examined with exact two-sided permutation tests on the difference in means over all 184,756 allocations, which target the estimand directly, and with exact two-tailed Mann–Whitney U tests, which test stochastic equality rather than the mean difference. Categorical variables were compared with the Fisher–Freeman–Halton exact test. Consistent with the estimation framework of this pilot (Section 2.1), point estimates and intervals carry the interpretation and *p* values are descriptive; no adjustment for multiplicity was made, since component-level analyses were exploratory.

All analyses treat the procedure as the unit of observation. Because operators were nested within arms and operator identity was not recorded, clustering could not be modeled; the effective sample size is therefore smaller than 20, and the intervals for workflow time and the costs derived from it are likely too narrow. The same applies to residual alcoholic chlorhexidine in the bulk-bottle arm, which depends on the volume each operator decanted, to aqueous chlorhexidine discarded after the pre-scrub, and to total measured waste, which includes both. It does not apply to material cost and solid waste, which are fixed by each protocol’s inventory.

Post hoc sensitivity analysis of case mix. Because procedure types were unevenly distributed between arms (Table 3) and prepared field size was not recorded, we repeated the principal comparisons after restricting both arms to unilateral hernia repair, the only procedure type represented by more than one case in each arm (7 applicator and 4 bulk-bottle procedures). This analysis was not prespecified and was undertaken during peer review. Outcomes were total workflow time, residual alcoholic chlorhexidine, total measured waste, total base-case cost, and two components of workflow time: the steps absent from the applicator workflow (decanting onto a cloth with application, and removal of excess with gauze) and the steps common to both workflows. With four observations in one arm, the BCa acceleration constant cannot be estimated reliably, so intervals were obtained by the percentile method (10,000 resamples stratified by delivery system, seed = 2,000,000). We also report exact two-sided permutation tests on the difference in means, enumerating all 330 possible allocations of the 11 procedures; the smallest attainable *p* value is therefore 0.003. To examine whether prepared field size rather than delivery format accounted for the differences, we compared, within the bulk-bottle arm only, procedures with a large prepared field (bilateral hernia, incisional hernia and HIPEC; *n* = 5) with unilateral hernia repair (n = 4), using exact permutation tests over all 126 allocations. Procedure type and the two workflow-time components are included in the deposited dataset [38].

Analyses were performed in IBM SPSS Statistics 30 (IBM Corp., Armonk, NY, USA). The dataset, variable dictionary and SPSS syntax are openly available in Zenodo [38]. Supplementary figures were produced with Matplotlib 3.10 (Python 3.12) [39]. The study is reported according to the STROBE and CHEERS 2022 statements (Supplementary Tables S1 and S2).

## 3. Results

### 3.1. Results—Piloting Phase I

The pilot phase (inguinal and umbilical hernia repair and cholecystectomy) showed that skin antisepsis did not consistently follow the IQZ back-and-forth technique [28,29,40], which limited comparability between procedures; staff were therefore trained in that technique before the comparative phase. It also led to three refinements of waste measurement: packaging was weighed, bulk-bottle consumption was measured as the change in bottle weight, and the residual weight of emptied bottles was included.

### 3.2. Basal Characteristics Phase II

All 20 procedures met the eligibility criteria and were analyzed, with no interrupted observations or missing data. Table 3 shows the procedures by delivery system. Their distribution did not differ significantly, but the sample has little power to detect imbalance: the bulk-bottle arm included the procedures with the largest prepared fields, and procedure type is only a weak proxy for field size, which was not recorded (Section 3.6).

### 3.3. Comparisons of Bulk Bottle and Applicator Methods Time

Table 4 summarizes workflow times by phase and component. The overall difference was concentrated in the preparatory phase and arose principally from two steps absent from the applicator workflow: pouring chlorhexidine onto a reusable cloth and removing excess solution with sterile gauze, together accounting for 39.8 s per procedure. Across the steps performed in both arms, point estimates instead favored the bulk-bottle protocol by 10.0 s. Component-level comparisons were exploratory and were not adjusted for multiplicity.

**Figure 1:**
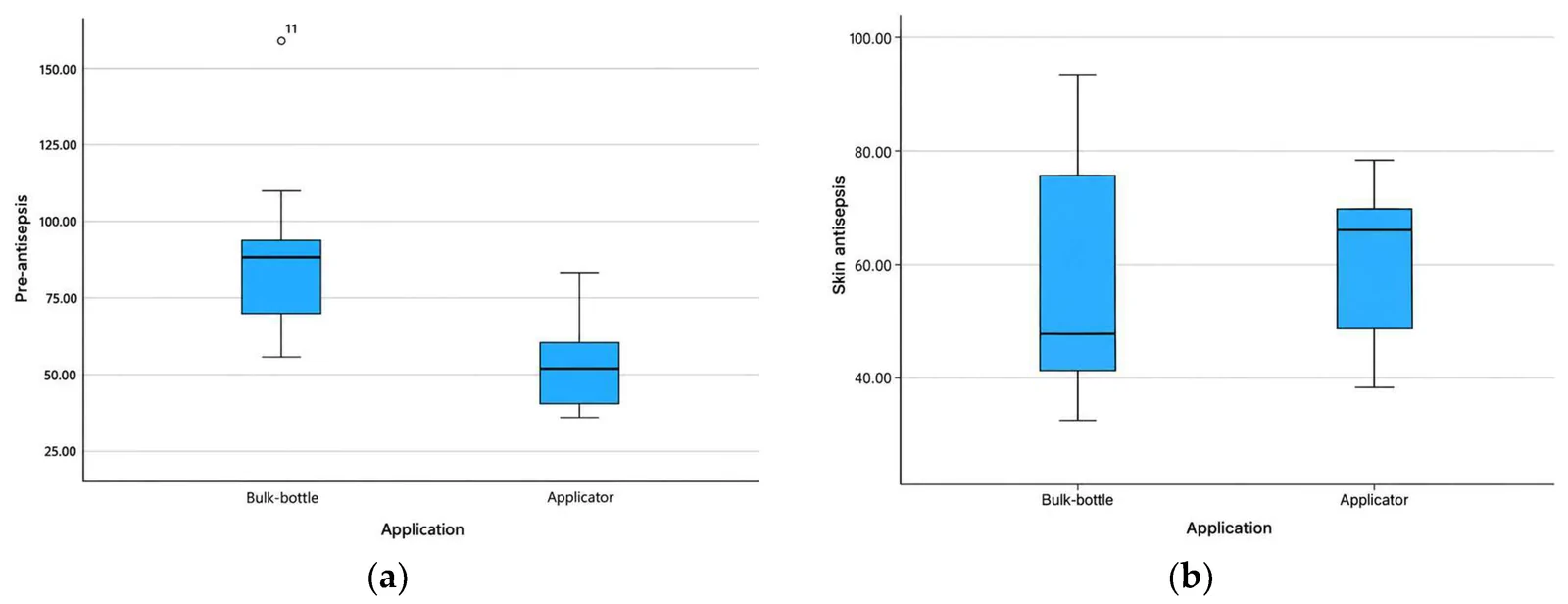
Time of application: (**a**) pre-antisepsis; (**b**) skin antisepsis.

Figure 1 illustrates the shift toward shorter pre-antisepsis times and the lower observed dispersion in the applicator arm. Variances were not formally compared, and the sequential design with non-overlapping operator groups precludes attributing the lower dispersion to the delivery system.

### 3.4. Comparisons of Bulk Bottle and Applicator Residuals

Table 5 summarizes waste outcomes. The difference was driven by residual alcoholic chlorhexidine and solid disposables; aqueous chlorhexidine discarded after the pre-scrub, a step common to both protocols, showed no clear difference. Solid waste excludes the dry mass of items weighed impregnated (17.00 g of gauze and the 41.12 g applicator device); had these been counted, solid waste would be 60.00 g and 68.12 g respectively, reversing this component, whereas residual antiseptic and total waste already include all discarded mass.

Residual antiseptic was also far less variable with the applicator, consistent with its fixed volume, whereas the quantity decanted from a bulk bottle varied case by case.

### 3.5. Economic Analysis

The incremental cost per procedure (applicator minus bulk bottle) was −€7.57 (BCa 95% CI −€14.38 to −€1.56; exact Mann–Whitney U *p* = 0.063; €2024) (Table 6). This is an accounting cost difference under the valuation described in Section 2.4 and, as detailed below, largely the workflow-time difference expressed in monetary units; among comparable procedures it fell to −€2.74 and could not be distinguished from zero (Section 3.6).

Personnel and operating room components accounted for 85% of the total difference. They value released time as an opportunity cost, whereas only the material difference (−€1.17, or −€3.26 on the same regulatory basis) is expenditure avoided per procedure. Personnel cost, operating room cost and workflow time are the same contrast in three units, which is why they share their *p* values; total cost differs only by the fixed material term, which shifts the distribution without widening it and accounts for its smaller *p* values. The BCa limits nearest zero for the time-dependent components (−€0.27 and −€0.13) do not locate the boundary of the effect with useful precision (Section 2.5; Table 6).

Sensitivity, scenario and threshold analyses are reported in Supplementary Tables S4–S7 and Figures S1–S3. No ±20% variation in any single cost parameter reversed the direction of the result, personnel cost being the most influential parameter in Table S4; varying the reprocessing allocation alone by ±20% had a larger effect (range €2.16) without reversing it. Alternative staffing complements and the second hospital’s tariffs changed the magnitude but not the direction of the base-case difference, which reduced to the material term alone (−€1.17, or −€3.26 on the same regulatory basis) when both personnel and operating room time were assigned no opportunity value. Price thresholds were remote from the observed values: the applicator would have to cost €14.50 (€16.59 under the medicinal-grade scenario) before the difference reached zero. The material difference was, however, sensitive to the allocation of reprocessing costs (€5.41 per procedure; Table 2; Table S6, panel c): it reached zero when reprocessing cost fell by 21.6% (to €4.24; by 60.3% under the medicinal-grade scenario), and with a zero marginal reprocessing cost the applicator was €4.24 (€2.15) more expensive in materials. Workflow time was the decisive parameter. Break-even occurs when the applicator workflow is 5.4 s slower than the bulk-bottle workflow (15.2 s under the medicinal-grade scenario). The full-sample estimate (−29.8 s) lies 35.2 s from that threshold, whereas the estimate among comparable procedures (−7.3 s; 95% CI −33.0 to +19.6; Section 3.6) lies 12.7 s from it, and its interval includes it. All these analyses hold the full-sample time difference fixed and do not propagate its uncertainty.

### 3.6. Sensitivity Analysis Restricted to Comparable Procedures

Table 7 shows the comparison restricted to unilateral hernia repair. The differences attributable to the structure of the two workflows were preserved: the two steps absent from the applicator workflow accounted for 39.5 s, and residual alcoholic chlorhexidine was 46.7 mL lower and total measured waste 44.8 g lower with the applicator, each with the smallest *p* value attainable under exact permutation (*p* = 0.003). The aggregate differences in time and cost were not preserved. Total workflow time differed by −7.3 s (95% CI −33.0 to +19.6), against −29.8 s in the full sample, and total base-case cost by −€2.74 (95% CI −€8.26 to +€3.05), against −€7.57; both intervals include zero. Among these procedures the steps common to both workflows took 32.2 s longer with the applicator (95% CI +4.5 to +60.0; permutation *p* = 0.061), offsetting most of the structural saving. In the full sample the same contrast was +10.0 s, so the uneven distribution of procedure types had partly masked it. The longest single observation in the study, a bulk-bottle gastric bypass (253 s), falls outside both comparisons.

**Table 7:** Sensitivity analysis restricted to unilateral hernia repair (bulk bottle n = 4; applicator n = 7).

Outcome	Bulk-Bottle Mean	Applicator Mean	Δ (Applicator − Bulk)	Percentile 95% CI *	MWU Exact *p* ^†^	Permutation Exact *p* ^‡^
Total workflow time (s)	115.75	108.43	−7.32	−33.00, +19.64	0.648	0.597
Steps absent from the applicator workflow (s)	39.50	0.00	−39.50	−46.50, −35.50	n/a ^§^	0.003
Steps common to both workflows (s)	76.25	108.43	+32.18	+4.50, +60.04	0.109	0.061
Residual alcoholic chlorhexidine (mL)	65.06	18.37	−46.69	−85.28, −23.88	0.006	0.003
Total measured waste (g)	149.75	105.00	−44.75	−78.61, −13.64	0.006	0.003
Total cost, base case (€)	32.97	30.22	−2.74	−8.26, +3.05	0.527	0.406

* Stratified bootstrap (by delivery system), 10,000 resamples, percentile method, seed = 2,000,000; with four observations in the bulk-bottle arm, intervals are indicative only. ^†^ Mann–Whitney U, exact significance, two-tailed. ^‡^ Exact two-sided permutation test on the difference in means over all 330 allocations; the smallest attainable value is 0.003. ^§^ Zero in every applicator procedure by construction; no rank test is meaningful. Differences are computed from unrounded values. Post hoc analysis, not prespecified. Operators remain nested within arms, so this analysis bounds the contribution of case mix but does not remove operator confounding.

Within the bulk-bottle arm, procedures with a large prepared field took 27.9 s longer than unilateral hernia repairs (95% CI −3.8 to +61.4; *p* = 0.206) but no difference was detected in antiseptic consumption (−1.7 mL; 95% CI −43.7 to +33.5; *p* = 0.952) or waste (−2.8 g; 95% CI −44.4 to +36.2; *p* = 0.905) in this small comparison (five vs. four procedures), whose intervals do not exclude a meaningful difference. This is consistent with the hypothesis that, in the bulk-bottle protocol, residual antiseptic reflects the volume decanted rather than the area prepared. Case mix thus biased the aggregate time and cost estimates in favor of the applicator and accounted for most of their full-sample difference, but did not account for the waste findings. A breakdown by procedure type is provided in Table S3.

## 4. Discussion

### 4.1. Main Findings

In this pilot study, with the same alcoholic chlorhexidine in both arms, the principal difference between the two workflows was structural: two handling steps required by the bulk-bottle protocol (39.8 s combined) have no counterpart in a pre-filled applicator. This is the only result that neither operator nor case mix can explain, because both steps are absent from every applicator procedure by construction. Eliminating transfer from a container is inherent to the applicator, whereas the ancillary items and their reprocessing reflect our center’s protocol, so a leaner bulk protocol would narrow the difference.

The applicator workflow was also associated with less residual alcoholic chlorhexidine (−72.8%) and total measured waste (−27.9%), differences that persisted among comparable procedures. Within the bulk-bottle arm, larger prepared fields took longer, but no difference in antiseptic consumption was detected in this small comparison (five vs. four procedures); we hypothesize that residual antiseptic in a bulk protocol is governed by the volume decanted rather than by the area prepared, which a confirmatory study recording field size could test.

The aggregate differences in time (−29.8 s) and cost (−€7.57) did not persist: among unilateral hernia repairs they fell to −7.3 s and −€2.74, with intervals including zero, because the applicator arm took 32.2 s longer on the shared steps. This offset cannot be attributed to the device, since operators were nested within arms, and whether the structural saving becomes a net saving is the central question for a confirmatory study. The cost difference is largely the time difference valued at unit rates (85% time-dependent) and inherits its uncertainty and bias. The component independent of time valuation, €1.17 per procedure (€3.26 on the same regulatory basis), depends on the allocation of reprocessing costs and reverses if reprocessing is valued below about 78% of its average allocated cost (Section 3.5 and Section 4.4).

### 4.2. Comparison with Previous Studies

With the same chlorhexidine–alcohol formulation, applicator and gauze application have shown comparable antimicrobial performance, and the advantages in standardization, time and resources attributed to the applicator were inferred rather than measured [15]; the present study measures them directly. Conversely, an applicator left more unprepared skin than gauze when preparing the hand [18]; because coverage was not assessed here, our findings are not evidence of better skin preparation. Our pilot phase also showed inconsistent adherence to the IQZ technique before training [28,29,40]. Standardizing it in both arms prevented the comparison from conflating delivery format with technique, consistent with evidence that application method affects performance and adherence [14,16,41,42,43,44]. The 30 s back-and-forth technique was used because the IQZ protocol and the instructions for use specify it for abdominal sites [27,29], not because it has been shown to be superior to concentric application.

### 4.3. Waste and Environmental Implications

NICE links its recommendation of alcohol-based chlorhexidine with a responsibility to consider environmental impact [5]. A life-cycle assessment reported about 49% lower CO_2_-equivalent emissions for applicator preparation than for bulk antiseptic with reusable instruments, with sterilization and unused decanted antiseptic as major contributors [26], and direct observation in other surgical settings has shown that unused supplies account for a measurable share of procedural material cost [45]. Our data provide a directly measured estimate of the unused-antiseptic component with the antiseptic agent held constant. They do not show a general environmental advantage of single-use devices: the balance varies with product and reprocessing context [24,25], and we measured waste at the point of care only, not manufacturing, reprocessing energy and water, or disposal [19,20].

Waste in the applicator arm depends on matching applicator size to the prepared field, just as waste in the bulk arm depends on the volume decanted. With the 26 mL format, about 70% of the nominal fill (18.05 of 26 mL) was not delivered to the skin in procedures that were mostly unilateral hernia repairs; a smaller format matched to the field could reduce this residual and its material cost, provided coverage is not compromised. In both arms, residual antiseptic therefore reflects a choice of volume rather than a fixed property of the delivery system.

### 4.4. Economic Interpretation

Under average-cost allocation of reprocessing, the material component favored the applicator even though the applicator cost more than the bulk antiseptic it replaced, because the bulk protocol required ancillary items and their reprocessing. Headline price comparisons can therefore mislead: a recent review contrasted a £8.46 applicator with £1.73 for 150 mL of generic chlorhexidine–alcohol [5,23], although the NICE economic model itself included ancillary equipment and sterilization. Our own material result, however, depends on how reprocessing is costed. Average tray-space shares approximate long-run marginal cost where sterilization capacity is constrained and reusable items displace other loads; where they are added to containers that would be sterilized anyway, the short-run marginal cost of reprocessing may be much lower, and the material difference reverses once reprocessing is valued below about 78% of its allocated cost (Section 3.5). The sign of the only component that represents avoided expenditure therefore depends on local reprocessing capacity. The relevant comparison is between locally observed procedure costs and local break-even prices. Most of the accounting difference, however, reflects released time, whose value depends on the local opportunity value of operating room capacity and which, among comparable procedures, lay within the range that would reverse the result (Section 3.5). The transferable element is therefore the model rather than the absolute estimate: quantities and unit costs are reported separately, and an open spreadsheet accepts local times, prices and tariffs [46]. Unlike previous economic comparisons, which changed the antiseptic agent together with the delivery format [21] or depended on assumptions about infection costs [22], this provider-perspective micro-costing isolates the resources consumed by the two workflows; evidence of a difference in infection risk would alter that balance.

### 4.5. Limitations

The principal limitation is the non-randomized pilot design. No surgeon used both systems, so operator and device effects cannot be separated, and because operator identity was not recorded, clustering could not be modeled and the intervals for workflow time, time-dependent costs and liquid and total waste are likely too narrow. The structural difference is exempt from this concern; the shared steps, which were slower in the applicator arm, are not. Case mix was the second source of bias. Prepared area, body habitus and delivered volume were not recorded, and the restricted analysis (Section 3.6) showed that case mix accounted for about three quarters of the time difference and nearly two thirds of the cost difference, but not for the waste differences. That analysis rests on four bulk-bottle procedures and remains subject to operator confounding, so it bounds rather than removes the contribution of case mix.

Measurement was not blinded, and a single observer timed all procedures without duplicate timing. Staff were aware of being observed, which may have altered their performance in both arms. The arms were observed sequentially, 26 days apart, so temporal changes and observer learning are confounded with the delivery system; learning would mainly reduce variability in the arm observed second, the applicator arm, and could contribute to its lower dispersion, but it is unlikely to affect the structural steps or the weighed waste.

The findings apply to elective abdominal surgery performed with a standardized technique and to the study center’s bulk-bottle protocol, whose reprocessing costs were allocated by an internal tray-space share that has not been externally validated and reflects average rather than short-run marginal cost (Section 4.4); centers with disposable or fewer ancillary items, other teams or other field sizes could observe different differences. What may transfer is the mechanism rather than the absolute estimates.

Only operational and economic endpoints were assessed. Antimicrobial effectiveness, residual skin chlorhexidine and SSIs were not evaluated, and the findings are conditional on the antimicrobial equivalence of application methods reported elsewhere [15]. Workflow time excluded the air-drying interval, which may differ between systems with the volume of alcohol left on the skin [17], so the observed difference cannot be equated with time to draping or incision.

Time was valued with aggregate institutional tariffs, and the base case assumed senior staffing, which favors the faster workflow; alternative rates and a second hospital’s tariffs did not change the direction of the result, but both sites belong to the same institution. Economic uncertainty was explored with deterministic analyses, which do not propagate joint parameter uncertainty as a probabilistic analysis would [47,48,49].

The study was funded by the manufacturer of the applicator evaluated, and the funder defined its objectives: the assessment of workflow time, waste and per-procedure cost. Although the funder had no access to the data and did not review the manuscript, the outcomes reflect that scope, and endpoints on which an applicator might perform less well, such as coverage of the prepared area, were not included. One author received personal fees from the funder for presenting preliminary results. The findings should be read with this in mind; the deposited dataset and syntax allow the reported estimates to be reproduced independently.

## 5. Conclusions

In this pilot study, with the same alcoholic chlorhexidine in both arms, a pre-filled applicator removed two handling steps required by the study center’s bulk-bottle protocol and was associated with substantially less residual antiseptic and total measured waste, differences that were not explained by case mix. Among comparable procedures, slower execution of the shared steps offset the structural saving, so total workflow time and per-procedure cost did not differ detectably; the larger full-sample differences were biased in favor of the applicator by case mix. The difference in material cost, €1.17 to €3.26 per procedure, depends on how reprocessing is costed and reverses when its marginal cost is low. Whether the structural saving becomes a net saving in time or cost therefore remains open. A confirmatory study should allocate both systems within operators, record operator identity, prepared field size and the volume of antiseptic delivered, measure drying time and time to draping, and assess coverage of the prepared area alongside workflow and waste.

## Figures and Tables

**Table 1:** Unit costs per minute used to value personnel and operating room time (€2024).

Resource	Number Engaged	Unit Cost * (€/min)	Subtotal (€/min)
Surgeon	2	1.63	3.26
Anesthesiologist	1	1.63	1.63
Scrub nurse	1	1.20	1.20
Circulating nurse	1	1.20	1.20
Anesthesia nurse	1	1.20	1.20
**Personnel subtotal**			8.49
Operating room (facilities and equipment)	—	4.40	4.40
**Combined rate**			12.89

Bold formatting is used to distinguish subtotals and totals from individual items. * Fully loaded institutional rates (gross salary, employer social security contributions, liability insurance and contractual benefits). A single rate was applied to surgeons and the anesthesiologist, and a single nursing rate to all operating room nurses.

**Table 2:** Item-level material costs (€2024) used in the micro-costing analysis, by skin-antisepsis system.

Cost Item	Basis for Calculation	Bulk-Bottle, Base Case (€)	Bulk-Bottle, Sensitivity Scenario (€) *	Applicator (€)
Alcoholic chlorhexidine 2% (biocide)	250 mL bottle (€3.32) apportioned across 3 procedures	1.11	—	—
Alcoholic chlorhexidine 2% (medicinal)	100 mL bottle (€3.20), single-use, one bottle per procedure	—	3.20	—
Single-use applicator	Single-use, pre-filled	—	—	6.93
Washcloth packaging		0.30	0.30	—
Washcloth reprocessing	€45 sterilization container × 3.5% tray-space share	1.58	1.58	—
Reusable gallipot (per use)	€4.20 unit cost ÷ 150 uses	0.03	0.03	—
Gallipot packaging		0.50	0.50	—
Gallipot reprocessing	€45 × 5% tray-space share	2.25	2.25	—
Sterile gauzes + packaging	20 gauzes + packaging (€0.898) shared across 2 procedures	0.45	0.45	—
Gripper packaging		0.30	0.30	—
Gripper reprocessing	€45 × 3.5% tray-space share	1.58	1.58	—
Total material cost per procedure		8.10	10.19	6.93

* Sensitivity scenario in which the bulk-bottle arm is costed with a medicinally licensed chlorhexidine–alcohol formulation, so that both arms are valued on the same regulatory basis. The washcloth used after the aqueous pre-scrub, common to both arms, is not costed in either.

**Table 3:** Distribution of type of surgical intervention by delivery system.

Disinfection Application	Applicator n (%)	Bulk bottle n (%)	Total n (%)
Surgical Intervention ^†^			
Bilateral hernia	0 (0%)	3 (30%)	3 (15%)
Gall bladder	1 (10%)	0 (0%)	1 (5%)
Gastric bypass	1 (10%)	1 (10%)	2 (10%)
Hernia	7 (70%)	4 (40%)	11 (55%)
HIPEC	0 (0%)	1 (10%)	1 (5%)
Incisional hernia	0 (0%)	1 (10%)	1 (5%)
Pancreas	1 (10%)	0 (0%)	1 (5%)

^†^ Fisher–Freeman–Halton exact test. HIPEC = Hyperthermic intraperitoneal chemotherapy, also known as Sugarbaker.

**Table 4:** Comparison of time spent (in seconds) across phases of skin antisepsis using bulk-bottle versus applicator methods.

Phase	Bulk-Bottle Mean (SD)	Applicator Mean (SD)	Δ (Applicator − Bulk)	BCa Bootstrap 95% CI *	Percentile 95% CI	Permutation *p* ^¶^	MWU Exact *p* ^†^
**Pre-antisepsis phase (subtotal)**	89.30 (29.48)	53.30 (14.94)	−36.00	−56.40, −18.10	−56.90, −17.80	0.0009	0.001
Material preparation	9.70 (0.95)	7.00 (0.00)	−2.70	−2.70, −2.10 ^§^	−2.70, −2.10	0.0001	<0.001
Scrubbing the patient with CHG (40 mg/mL)	35.00 (21.97)	35.10 (13.53)	+0.10	−16.30, +14.80	−16.10, +14.50	1.000	0.684
Chlorhexidine excess removal with a washcloth	15.70 (5.83)	11.20 (3.68)	−4.50	−8.30, −0.60	−8.50, −0.40	0.061	0.089
Pouring chlorhexidine into a reusable cloth & application	28.90 (9.26)	0 (0)	−28.90	n/a ^‡^	n/a ^‡^	<0.0001	n/a ^‡^
**Skin antisepsis phase (subtotal)**	54.10 (20.39)	60.30 (13.78)	+6.20	−9.00, +20.50	−8.80, +20.20	0.439	0.353
Back-and-forth application (B&F)	43.20 (21.61)	60.30 (13.78)	+17.10	+0.90, +32.40	+1.60, +31.80	0.052	0.043
Removal of chlorhexidine excess with a single sterile gauze	10.90 (3.25)	0 (0)	−10.90	n/a ^‡^	n/a ^‡^	<0.0001	n/a ^‡^
Total time	143.40 (48.29)	113.60 (21.56)	−29.80	−61.40, −1.90	−63.10, −0.60	0.085	0.143
Steps absent from the applicator workflow	39.80 (8.26)	0 (0)	−39.80	−44.40, −35.50	−44.90, −35.20	<0.0001	n/a ^‡^
Steps common to both workflows	103.60 (45.00)	113.60 (21.56)	+10.00	−20.16, +37.40	−21.00, +38.00	0.547	—

Bold values indicate phase subtotals and overall totals. * Stratified bootstrap (by delivery system), 10,000 resamples, seed = 2,000,000; BCa and percentile intervals are computed from the same resamples. ^†^ Mann–Whitney U, exact significance, two-tailed. ^‡^ Zero in every applicator procedure by construction; the difference is structural rather than empirical, so no rank test is reported, and no interval for the individual steps. The permutation *p* value reflects this deterministic contrast. ^§^ BCa correction completed on 6533 of 10,000 resamples, because the applicator arm had zero variance at this step; the interval is retained as a descriptive summary, and its lower limit coincides with the point estimate for that reason. ^¶^ Exact two-sided permutation test on the difference in means over all 184,756 allocations. Intervals assume independent observations; because operators were nested within arms and operator identity was not recorded, they are likely narrower than the true sampling uncertainty. Phase subtotals (pre-antisepsis; skin antisepsis) are the sums of the components listed beneath them: in the bulk-bottle arm the skin antisepsis phase comprises back-and-forth application and removal of excess with gauze, and in the applicator arm back-and-forth application only. Total time is the sum of the two phase subtotals, which equals the sum of all components; no row is counted twice.

**Table 5:** Comparison of procedure-related waste using bulk-bottle versus applicator methods.

Waste Component	Bulk-Bottle Mean (SD)	Applicator Mean (SD)	Δ (Applicator − Bulk)	Relative Change	BCa Bootstrap 95% CI *	Percentile 95% CI	Permutation *p* ^¶^	MWU Exact *p* ^†^
Liquid waste, alcoholic chlorhexidine (mL)	66.36 (29.54)	18.05 (2.57)	−48.32	−72.8%	−65.93, −32.18	−66.50, −31.84	<0.0001	<0.001
Liquid waste, aqueous chlorhexidine from pre-scrub (mL)	33.00 (11.89)	25.10 (11.55)	−7.90	−23.9%	−17.60, +2.20	−17.60, +2.20	0.157	0.063
Solid waste, dry disposables and packaging (g)	43.00 (0.00)	27.00 (0.00)	−16.00	−37.2%	n/a ^‡^	n/a ^‡^	n/a ^‡^	n/a ^‡^
Total measured waste (g)	151.40 (31.54)	109.10 (12.11)	−42.30	−27.9%	−62.10, −22.60	−62.10, −22.60	0.0009	<0.001

* Stratified bootstrap (by delivery system), 10,000 resamples, seed = 2,000,000; BCa and percentile intervals are computed from the same resamples. ^†^ Mann–Whitney U, exact significance, two-tailed. ^‡^ The protocol fixed solid waste and was identical in every procedure within each arm; both standard deviations are zero, so no t statistic can be computed, and the rank test reflects perfect separation by design rather than empirical sampling variability. The difference is a deterministic consequence of the material inventory and no interval or significance test is reported. Total measured waste includes the mass retained by impregnated items and therefore exceeds the sum of the rows above. ^¶^ Exact two-sided permutation test on the difference in means over all 184,756 allocations. Intervals assume independent observations; because operators were nested within arms and operator identity was not recorded, they are likely narrower than the true sampling uncertainty. Differences are computed from unrounded values; the rounded means for alcoholic chlorhexidine give −48.31 mL.

**Table 6:** Per-procedure costs (€2024) for the bulk-bottle and applicator systems, base case and medicinal-grade scenario.

Cost Component	Bulk-Bottle Mean (SD)	Applicator Mean (SD)	Δ (Applicator − Bulk)	BCa Bootstrap 95% CI *	Percentile 95% CI	Permutation *p* ^¶^	MWU Exact *p* ^†^
***Base case (biocide-registered bulk product)***							
Material (€)	8.10 (0.00)	6.93 (0.00)	−1.17	n/a ^‡^	n/a ^‡^	n/a ^‡^	n/a ^‡^
Personnel (€)	20.29 (6.83)	16.07 (3.05)	−4.22	−8.69, −0.27	−8.93, −0.09	0.085	0.143
Operating room (€)	10.52 (3.54)	8.33 (1.58)	−2.19	−4.51, −0.13	−4.63, −0.04	0.085	0.143
**Total cost (€)**	38.91 (10.37)	31.34 (4.63)	**−7.57**	−14.38, −1.56	−14.73, −1.30	0.038	0.063
*Medicinal-grade scenario (100-mL bottle, €3.20, single use)* **^^§^^**							
Material (€)	10.19 (0.00)	6.93 (0.00)	−3.26	n/a ^‡^	n/a ^‡^	n/a ^‡^	n/a ^‡^
**Total (€)**	41.00 (10.37)	31.34 (4.63)	**−9.66**	−16.47, −3.65	−16.82, −3.39	0.007	0.012

Scenario headings are shown in italics, and total costs are shown in bold. SD = standard deviation; BCa = bias-corrected and accelerated; MWU = Mann–Whitney U. * Stratified bootstrap (by delivery system), 10,000 resamples, seed = 2,000,000; BCa and percentile intervals are computed from the same resamples. ^†^ Mann–Whitney U, exact significance, two-tailed. ^‡^ Material cost is a fixed per-item accounting input (unit price × quantity) with zero within-group variance; an interval or significance test would reflect the deterministic price assignment rather than empirical sampling variability, and is therefore not reported. ^§^ Only the material term changes; personnel and operating room costs are identical to the base case. ^¶^ Exact two-sided permutation test on the difference in means over all 184,756 allocations. Personnel and operating room cost are the workflow-time comparison of Table 4 expressed in monetary units and share its *p* values. Intervals assume independent observations; because operators were nested within arms and operator identity was not recorded, they are likely narrower than the true sampling uncertainty. The base case assumes senior medical staffing, the valuation most favorable to the faster workflow; alternative staffing scenarios are reported in Supplementary Table S5.

## Data Availability

The analysis dataset supporting the reported findings (procedure-level workflow times, waste measurements and costed components for the 20 observed procedures), together with the variable dictionary, the SPSS syntax and the Python script reproducing all reported analyses, is openly available in Zenodo at https://doi.org/10.5281/zenodo.23024432 (version 2.0.0), under a CC BY 4.0 license. The resource-use and cost model underlying the reported estimates is openly available in Zenodo at https://doi.org/10.5281/zenodo.22101863; that identifier resolves to the most recent version of the model. The data contain no patient identifiers and no patient-level demographic or clinical information, since none were collected: the unit of observation is the procedure, not the patient. The medicinal-grade scenario reported in Table 6 is a re-costing of the same 20 observed procedures at an alternative unit price, not an additional set of observations.

## Supplementary Materials

The following supporting information can be downloaded at https://www.mdpi.com/article/10.3390/healthcare14203456/s1: Section S1. Waste definitions and weighing procedures; Section S2. Allocation of reprocessing costs; Table S1. STROBE Statement—checklist of items that should be included in reports of observational studies; Table S2. CHEERS 2022 checklist; Table S3. Outcomes by procedure type and delivery system; Table S4. One-way sensitivity analysis: effect of ±20% variation in each cost parameter on the incremental cost per procedure (applicator − bulk bottle), €2024; Table S5. Scenario analysis: effect of alternative staffing complements and institutional tariffs on the incremental cost per procedure (applicator − bulk bottle), €2024; Table S6. Threshold (break-even) analysis: value each parameter would have to reach for the incremental cost (panels a and b) or the material cost difference (panel c) to fall to zero, all other parameters held at base-case values; Table S7. Two-way sensitivity analysis: incremental cost per procedure (applicator − bulk bottle, €2024) across combinations of the combined operating room + personnel rate and the applicator unit price. Negative values favor the applicator; Figure S1. One-way sensitivity analysis of the incremental cost per procedure (applicator − bulk bottle), €2024. Each bar shows the effect of varying a single parameter by ±20% while holding all others at base-case values. The vertical red line marks the base-case incremental cost. (a) Bulk-bottle arm using biocide-registered chlorhexidine; (b) bulk-bottle arm using medicinal-grade chlorhexidine; Figure S2. Threshold analysis of the incremental cost per procedure (applicator − bulk bottle), €2024. Each panel varies one parameter across a plausible range while holding all others at base-case values; the horizontal line at zero marks break-even and circles mark the break-even value. Dotted vertical lines and squares indicate observed base-case values. (a) Applicator unit price; (b) bulk-bottle material cost per procedure; (c) workflow time difference between systems; Figure S3. Two-way sensitivity analysis of the incremental cost per procedure (applicator − bulk bottle), €2024, across the combined operating room plus personnel rate (*x*-axis) and the applicator unit price (*y*-axis). Blue shading denotes a lower cost per procedure with the applicator and red shading a higher cost; the solid black line is the break-even contour (incremental cost = €0), and the dotted lines are additional iso-cost contours labeled in euros. The white circle marks the observed base-case combination (€12.89 per minute; €6.93 per applicator). (a) Bulk-bottle arm costed with biocide-registered chlorhexidine (€8.10 per procedure); (b) bulk-bottle arm costed with medicinal-grade chlorhexidine (€10.19 per procedure); File S1. Delivery-format resource and cost model (spreadsheet implementation with a comma-separated file of the default parameters). File S1 is a frozen copy of version 1.3.0 of the model; the maintained version is archived in Zenodo at https://doi.org/10.5281/zenodo.22101863.

## Abbreviations

The following abbreviations are used in this manuscript:

B&F	Back-and-forth (application technique)
BCa	Bias-corrected and accelerated (bootstrap)
CHEERS	Consolidated Health Economic Evaluation Reporting Standards
CHG	Chlorhexidine gluconate
HIPEC	Hyperthermic intraperitoneal chemotherapy
IQZ	Infección Quirúrgica Zero (Spanish Zero Surgical Infection program)
OR	Operating room
SSI	Surgical site infection
STROBE	Strengthening the Reporting of Observational Studies in Epidemiology
