# Supplementary material for: Applicator-Based Versus Bulk-Bottle Delivery of the Same Alcoholic Chlorhexidine: A Single-Center, Non-Randomized Pilot Micro-Costing Study of Two Surgical Skin-Antisepsis Workflows
Source: Healthcare (Basel). 2026 Oct 15;14(20):3456. doi: 10.3390/healthcare14203456 (PMC13704735; doi:10.3390/healthcare14203456)
Supplement: Supplementary file 1 [file healthcare-14-03456-s001.zip › healthcare-4564642-supplementary.pdf]

Supplementary Materials

Section S1. Waste definitions and weighing procedures

S1.1 Definitions

Solid waste (g): the dry mass of discarded non-liquid items weighed in dry condition, comprising the pre-scrub sponge, all packaging, and, in the bulk-bottle arm, the per-procedure share of the emptied bottle. Items weighed in impregnated form, namely the sterile gauzes used to blot excess solution in the bulk-bottle arm and the applicator device in the applicator arm, are not included in this component; their dry mass and their retained liquid are both captured in total measured waste. Reusable items (gallipot, gripper and washcloths) were returned for reprocessing rather than discarded and are excluded from all waste endpoints.

Liquid waste: Alcoholic chlorhexidine remaining at the end of antisepsis in any receptacle, the applicator, or absorbent materials. When alcoholic chlorhexidine was recorded as mass (g), it was converted to volume (mL) using a density of 0.88 g/mL. This conversion was used consistently for the alcoholic-liquid waste values reported in the analyses and tables.

S1.2. Bulk-bottle arm (alcoholic chlorhexidine 2%)

For each case, we obtained three measurements:

- Bottle delta (consumption): The bottle was weighed immediately before and after the surgical intervention; the difference was quantified, and the solution used was not counted as waste.
- Unused solution (liquid waste): alcoholic chlorhexidine remaining in the gallipot or retained in absorbent materials at the end of antisepsis was recorded as liquid waste. Every such item was weighed in dry condition before use and again after use, and the retained antiseptic was taken as the difference between the two weights, so that no dry material mass was at any point counted as antiseptic. This procedure was applied to the gallipot, the sterile gauzes used to blot excess solution, and the reusable washcloths. Two washcloths were used per procedure in this arm, one to remove excess aqueous chlorhexidine after the pre-scrub and one to transfer the alcoholic solution to the skin; the applicator arm used a single washcloth, for the first of these purposes only.
- Solid waste: packaging of sterile gauzes/swabs and any other non-liquid disposables were weighed and recorded as solid waste.

S1.3 Applicator arm

For the applicator, we leveraged the device's known masses: The dry device mass (without liquid) and the standard filled mass (with liquid) of the applicator were known a priori. After each case, we measured the applicator's post-use mass (with any residual liquid). We calculated unused chlorhexidine (liquid waste) as the post-use mass minus the dry device mass; we then converted the resulting mass to volume using a density of 0.88 g/mL. The dry device mass was 41.12 g, obtained by subtracting the nominal fill volume (26 mL at 0.88 g/mL) from the filled mass (64.00 g), and was applied identically to every case. The conceptual definition of unused antiseptic is therefore the same in both arms: antiseptic made available for the procedure that was not delivered to the skin, obtained in each case as a post-use weight less a dry reference

weight. The solid waste component for this arm comprised the pre-scrub sponge, the applicator packaging and the swabs; the dry mass of the device itself was weighed as part of the used applicator and is included in total measured waste. The used (delivered) volume can be derived as the standard filled mass minus the post-use mass; however, consistent with our waste endpoints, we attributed only the unused portion to liquid waste.

Per-procedure variables. For each procedure, we created:

(i) System (bulk bottle vs. applicator).

(ii) Solid waste (g).

(iii) Liquid waste, reported separately for alcoholic chlorhexidine (mL, converted from mass using 0.88 g/mL) and for aqueous chlorhexidine discarded during the pre-scrub step (mL).

(iv) Total waste (g), measured as the gross weight of all material discarded after the procedure.

These variables were used for all comparisons between systems. Because total waste was weighed as collected, it includes components not captured by the two categories above, such as the retained mass of the used applicator body and gauzes and washcloths saturated with antiseptic; therefore, it is not the arithmetic sum of the solid and liquid components.

Section S2. Allocation of reprocessing costs

Reusable ancillary items in the bulk-bottle arm (washcloths, gallipot and gripper) are reprocessed in the hospital sterilization service inside a standard container, whose reprocessing cost is €45 according to the analytical accounting. Each item was allocated the share of that cost corresponding to the fraction of the container's tray space it occupies:

Reprocessing cost per procedure = €45 × tray-space share of the item. The washcloth used to remove excess aqueous chlorhexidine after the pre-scrub is common to both arms and is costed in neither; only the washcloth used to transfer the alcoholic solution in the bulk-bottle arm is costed, together with its packaging.

Washcloths: €45 × 3.5% = €1.58. Gallipot: €45 × 5% = €2.25. Gripper: €45 × 3.5% = €1.58. Total reprocessing cost per bulk-bottle procedure: €5.41.

Tray-space shares were estimated by the sterilization service and have not been externally validated. Because reprocessing accounts for €5.41 of the €8.10 bulk-bottle material cost, the material difference depends on how reprocessing is costed: it reaches zero when the reprocessing allocation falls by 21.6% (60.3% under the medicinal-grade scenario), and it reverses in a center using disposable ancillary items, or none, or where reprocessing is valued at a short-run marginal cost well below its average tray-space share, for example when the items are added to containers that would be sterilized anyway (Table S6, panel c). The effect of varying material prices and the reprocessing allocation is examined in Table S4, and break-even values in Table S6.

Table S1. STROBE Statement—checklist of items that should be included in reports of observational studies

	Item No.	Recommendation	Page No.	Relevant text from manuscript
**Title and abstract**	1	(*a*) Indicate the study's design with a commonly used term in the title or the abstract	Title; Abstract (Methods)	The title describes the study as a single-center, non-randomized pilot micro-costing study; the Methods of the Abstract state the prospective observational design.
		(*b*) Provide in the abstract an informative and balanced summary of what was done and what was found	Abstract; Highlights	Structured abstract reporting the design, the 20 procedures, the structural difference in handling steps, the waste and material-cost differences, the aggregate time and cost differences with 95% confidence intervals, and the case-mix sensitivity analysis with the direction of its bias.
**Introduction**				
Background/rationale	2	Explain the scientific background and rationale for the investigation being reported	Section 1	Burden of surgical site infection and guideline recommendations; distinction between antiseptic agent and delivery format; absence of direct evidence on the workflow, waste and cost consequences of the delivery format.

Objectives	3	State specific objectives, including any prespecified hypotheses	Section 1, final paragraph	To compare two complete skin-antisepsis workflows using the same alcoholic chlorhexidine (pre-filled applicator versus the study center's bulk-bottle protocol) in terms of workflow time, waste and per-procedure cost. No prespecified hypotheses; the study was designed for estimation (Section 2.1).
**Methods**				
Study design	4	Present key elements of study design early in the paper	Section 2.1; Abstract (Methods)	Prospective observational pilot study: a pilot phase of 50 procedures to standardize technique and measurement, followed by a comparative phase of 20 procedures (10 per workflow).
Setting	5	Describe the setting, locations, and relevant dates, including periods of recruitment, exposure, follow-up, and data collection	Section 2.1	Single Spanish university hospital. Pilot phase February–March 2024. Comparative phase: bulk-bottle procedures observed on 3, 9 and 10 April 2024 and applicator procedures on 6, 7 and 8 May and 12 June 2024 (sequential observation, 26 days apart).
Participants	6	(*a*) *Cohort study*—Give the eligibility criteria, and the sources and methods of selection of participants. Describe methods of follow-up<br>*Case-control study*—Give the eligibility criteria, and the sources and methods of case ascertainment and control selection. Give the rationale for the choice of cases and controls	Section 2.1	Elective abdominal general surgery in adults in whom 2% alcoholic chlorhexidine

		Cross-sectional study—Give the eligibility criteria, and the sources and methods of selection of participants		was indicated as routine care; exclusion criteria: chlorhexidine allergy and urgent or emergency surgery. Consecutive enrollment, as observer availability allowed, until 10 procedures per arm. No follow-up (intraoperative outcomes).
		(*b*) *Cohort study*—For matched studies, give matching criteria and number of exposed and unexposed<br>*Case-control study*—For matched studies, give matching criteria and the number of controls per case	Not applicable	The study was not matched. Allocation followed each surgeon's routine protocol; there was no randomization or matching.
Variables	7	Clearly define all outcomes, exposures, predictors, potential confounders, and effect modifiers. Give diagnostic criteria, if applicable	Sections 2.1–2.4; Table 3	Exposure: delivery workflow (pre-filled applicator versus bulk bottle), with antiseptic, concentration and back-and-forth technique held constant. Outcomes: workflow time by step and in total, excluding air drying (Section 2.2); residual alcoholic chlorhexidine, aqueous pre-scrub solution, solid and total measured waste (Section 2.3; Supplementary Section S1); per-procedure cost (Section 2.4). Potential confounders: operator (nested within arms, identity not recorded) and case mix (procedure

				type as a proxy for field size; Table 3).
Data sources/ measurement	8*	For each variable of interest, give sources of data and details of methods of assessment (measurement). Describe comparability of assessment methods if there is more than one group	Sections 2.2–2.4; Tables 1 and 2; Supplementary Sections S1 and S2	Workflow times by stopwatch against predefined intervals; residues weighed on a calibrated electronic balance; unit costs from the Hospital Pharmacy price list and the Analytical Accounting and Profitability Department. The same observer, instruments and definitions were used in both arms. A single observer timed all procedures, so inter-observer agreement could not be assessed (Section 4.5).
Bias	9	Describe any efforts to address potential sources of bias	Sections 2.1–2.5, 3.6 and 4.5; Funding	Pilot phase to fix the protocol, timing intervals and weighing procedures before comparative data collection; standardized technique in both arms; identical instruments and definitions; common eligibility criteria with no exclusions. Post hoc case-mix sensitivity analysis restricted to comparable procedures, quantifying the direction and size of case-mix bias (Sections 2.5 and 3.6). Senior-staffing

				valuation in the base case, the assumption most favorable to the faster workflow, with alternatives in Table S5. Residual biases (operator nesting, sequential observation and observer learning, absence of blinding) and their likely direction are discussed in Section 4.5; the role of the funder is stated after the Abstract and in Section 4.5.
Study size	10	Explain how the study size was arrived at	Section 2.1	No formal sample size calculation; the sample size was set by the number of procedures that could be observed in full without interfering with operating room activity. The pilot was designed to estimate effects and their variability in order to power a confirmatory study.

Continued on next page

Quantitative variables	11	Explain how quantitative variables were handled in the analyses. If applicable, describe which groupings were chosen and why	Sections 2.2–2.5	Times, weights, volumes and costs were analyzed as continuous variables and not categorized. Procedure type was used as recorded.
Statistical methods	12	(*a*) Describe all statistical methods, including those used to control for confounding	Section 2.5	Differences in means with BCa and percentile bootstrap 95% confidence intervals (10,000 resamples stratified by arm, seed 2,000,000), exact permutation tests on the mean difference and exact Mann–Whitney U tests; Fisher–Freeman–Halton exact test for procedure type. No adjustment for confounding, in keeping with the pilot design; confounding by case mix was examined by restriction (item 12e). IBM SPSS Statistics 30; percentile intervals verified in Python (SciPy).
		(*b*) Describe any methods used to examine subgroups and interactions	Sections 2.5 and 3.6; Table 7; Table S3	Post hoc analysis restricted to unilateral hernia repair (7 vs 4) and within-arm comparison of large versus small prepared fields in the bulk-bottle arm, with exact permutation tests. Outcomes by procedure type are reported descriptively (Table S3). Component-level comparisons in Table 4 are exploratory and not adjusted for multiplicity.

		(*c*) Explain how missing data were addressed	Section 3.2	There were no missing data; no imputation was required.
		(*d*) *Cohort study*—If applicable, explain how loss to follow-up was addressed<br>*Case-control study*—If applicable, explain how matching of cases and controls was addressed<br>*Cross-sectional study*—If applicable, describe analytical methods taking account of sampling strategy	Not applicable	Observation was confined to the intraoperative skin-antisepsis step; there was no follow-up.
		(*e*) Describe any sensitivity analyses	Sections 2.4, 2.5, 3.5 and 3.6; Tables 6 and 7; Tables S4–S7	Case-mix sensitivity analysis (Table 7); medicinal-grade regulatory scenario (Table 6); one-way ±20% analyses (Table S4); staffing and tariff scenarios, including a second hospital of the same institution and a zero valuation of time (Table S5); threshold analyses (Table S6); two-way analysis of applicator price and combined time rate (Table S7). Scenario estimates are deterministic recalculations from the observed mean times.
**Results**				
Participants	13*	(a) Report numbers of individuals at each stage of study—eg numbers potentially eligible, examined for eligibility, confirmed eligible, included in the study, completing follow-up, and analysed	Section 3.2	Twenty procedures met the eligibility criteria and were observed (10 per arm); none was excluded, interrupted or discarded, and all 20 were analyzed.
		(b) Give reasons for non-participation at each stage	Section 3.2	There was no non-participation at any stage.
		(c) Consider use of a flow diagram	Section 3.2	No flow diagram; with no exclusions or losses, the flow is reported narratively.

Descriptive data	14*	(a) Give characteristics of study participants (eg demographic, clinical, social) and information on exposures and potential confounders	Section 3.2; Table 3	Distribution of procedure type by arm (Fisher–Freeman–Halton exact test). No patient-level demographic or clinical data were collected; the unit of observation was the procedure.
		(b) Indicate number of participants with missing data for each variable of interest	Section 3.2	No missing data for any variable of interest.
		(c) *Cohort study*—Summarise follow-up time (eg, average and total amount)	Not applicable	There was no follow-up period.
Outcome data	15*	*Cohort study*—Report numbers of outcome events or summary measures over time	Sections 3.3–3.6; Tables 4–7	Summary measures for every outcome: workflow time by step and in total (Table 4), residual antiseptic and waste (Table 5), per-procedure costs for the base case and the medicinal-grade scenario (Table 6), and the restricted analysis (Table 7).
		Case-control study—Report numbers in each exposure category, or summary measures of exposure	Not applicable	This variant of the item does not apply.
		Cross-sectional study—Report numbers of outcome events or summary measures	Not applicable	This variant of the item does not apply.
Main results	16	(*a*) Give unadjusted estimates and, if applicable, confounder-adjusted estimates and their precision (eg, 95% confidence interval). Make clear which confounders were adjusted for and why they were included	Sections 3.3–3.6; Tables 4–7	All estimates are unadjusted mean differences with BCa and percentile bootstrap 95% confidence intervals, exact permutation p values and exact Mann–Whitney U p values. Confounding by case mix is addressed by restriction (Table 7) rather than by adjustment.

		(*b*) Report category boundaries when continuous variables were categorized	Not applicable	No continuous variable was categorised.
		(*c*) If relevant, consider translating estimates of relative risk into absolute risk for a meaningful time period	Not applicable	No relative risk or other relative measure of clinical effect was estimated; the outcomes are operational and economic rather than event-based.
Other analyses	17	Report other analyses done—eg analyses of subgroups and interactions, and sensitivity analyses	Sections 3.5 and 3.6; Tables 6 and 7; Tables S3–S7; Figures S1–S3	Case-mix sensitivity analysis and within-arm field-size comparison (Section 3.6; Table 7), outcomes by procedure type (Table S3), regulatory scenario (Table 6), and deterministic sensitivity, scenario, threshold and two-way analyses (Tables S4–S7; Figures S1–S3).
**Discussion**				
Key results	18	Summarise key results with reference to study objectives	Section 4.1; Section 5	The primary finding is structural: two handling steps required by the bulk-bottle protocol (39.8 s) are absent with the applicator. Residual antiseptic (−72.8%) and total waste (−27.9%) were lower and robust to case mix; the aggregate time and cost differences were not robust to case mix and could not be distinguished from zero among comparable procedures.
Limitations	19	Discuss limitations of the study, taking into account sources of potential bias or imprecision. Discuss both direction and magnitude of any potential bias	Section 4.5	Non-randomized pilot design with operators nested within arms and operator identity unrecorded; case mix, quantified by the restricted analysis; absence of blinding and a

				single observer; sequential observation and possible observer learning; single-center setting and center-specific bulk protocol; unvalidated reprocessing allocation; exclusion of drying time; absence of clinical endpoints; institutional tariffs and senior-staffing base case; deterministic treatment of parameter uncertainty; funding by the manufacturer of the applicator.
Interpretation	20	Give a cautious overall interpretation of results considering objectives, limitations, multiplicity of analyses, results from similar studies, and other relevant evidence	Sections 4.1–4.5; Section 5	Interpretation is framed as estimation. The aggregate time and cost differences are presented as estimates with their intervals and as biased by case mix; the conclusions are limited to the structural and waste findings and to what a confirmatory study should measure.
Generalisability	21	Discuss the generalisability (external validity) of the study results	Sections 4.4 and 4.5	The findings apply to elective abdominal surgery with a standardized technique and to the study center's bulk-bottle protocol, and the absolute estimates should not be extrapolated; what may transfer is the mechanism. Quantities and unit costs are reported separately, and an open spreadsheet model (File S1) allows re-costing with local prices.
**Other information**				

Funding	22	Give the source of funding and the role of the funders for the present study and, if applicable, for the original study on which the present article is based	Funding statement after the Abstract; Funding section; Section 4.5	Funded by Becton Dickinson (grant ECOPACT), manufacturer of the applicator evaluated. The funder defined the study objectives, had no access to the data, did not review the manuscript before submission and had no role in the decision to publish. The article processing charge was covered by discount vouchers awarded to the authors for peer review. See also the Conflicts of Interest statement.

*Give information separately for cases and controls in case-control studies and, if applicable, for exposed and unexposed groups in cohort and cross-sectional studies.

Note: An Explanation and Elaboration article discusses each checklist item and gives methodological background and published examples of transparent reporting. The STROBE checklist is best used in conjunction with this article (freely available on the Web sites of PLoS Medicine at http://www.plosmedicine.org/, Annals of Internal Medicine at http://www.annals.org/, and Epidemiology at http://www.epidem.com/). Information on the STROBE Initiative is available at www.strobe-statement.org.

Table S2. CHEERS 2022 checklist

Husereau D, Drummond M, Augustovski F, de Bekker-Grob E, Briggs AH, Carswell C, Caulley L, Chaiyakunapruk N, Greenberg D, Loder E, Mauskopf J, Mullins CD, Petrou S, Pwu RF, Staniszewska S; CHEERS 2022 ISPOR Good Research Practices Task Force. Consolidated Health Economic Evaluation Reporting Standards 2022 (CHEERS 2022) Statement: Updated Reporting Guidance for Health Economic Evaluations. BMJ. 2022;376:e067975.
The checklist is Open Access distributed in accordance with the terms of the Creative Commons Attribution (CC BY 4.0) license. See: http://creativecommons.org/licenses/by/4.0/.

Item	No.	Guidance for Reporting	Reported in section
**TITLE**			
**Title**	1	Identify the study as an economic evaluation and specify the interventions being compared.	Title. Identifies the study as a single-center, non-randomized pilot micro-costing study and names the two workflows compared (applicator-based versus bulk-bottle delivery of the same alcoholic chlorhexidine).
**ABSTRACT**			
**Abstract**	2	Provide a structured summary that highlights context, key methods, results and alternative analyses.	Abstract (structured); Highlights. Reports the design and setting, the 20 procedures, the structural difference in handling steps, waste, the material-cost difference (co-primary result), the aggregate time and cost differences with 95% confidence intervals, and the case-mix sensitivity analysis with the direction of its bias.
**INTRODUCTION**			
**Background and objectives**	3	Give the context for the study, the study question and its practical relevance for decision making in policy or practice.	Section 1. Burden of surgical site infection and guideline recommendations; distinction between antiseptic agent and delivery format; absence of direct economic and environmental evidence on delivery format. The objective is stated in the final paragraph.
**METHODS**			
**Health economic analysis plan**	4	Indicate whether a health economic analysis plan was developed and where available.	Sections 2.1, 2.4 and 2.5. No separate health economic analysis plan was developed, registered or published. The perspective, cost components, unit-cost sources and measurement procedures were fixed during the pilot phase and applied unchanged in the comparative phase. The case-mix sensitivity analysis (Section 2.5) was post hoc, undertaken during peer review, and is declared as such.
**Study population**	5	Describe characteristics of the study population (such as age range, demographics, socioeconomic, or clinical characteristics).	Sections 2.1 and 3.2; Table 3; Table S3. Adults undergoing elective abdominal general surgery in whom 2% alcoholic chlorhexidine was indicated as routine care. Table 3 reports procedure type by arm and Table S3 outcomes by procedure type. No patient-level demographic data were collected; the unit of observation was the procedure.
**Setting and location**	6	Provide relevant contextual information that may influence findings.	Section 2.1. Operating rooms of a single Spanish university hospital. Pilot phase February–March 2024; comparative phase April–June 2024, with the

			two arms observed sequentially, 26 days apart. Unit costs are institution-specific (Section 2.4).
**Comparators**	7	Describe the interventions or strategies being compared and why chosen.	Sections 2.1, 2.3 and 2.4; Table 2. The study center's bulk-bottle protocol (2% alcoholic chlorhexidine poured onto a reusable cloth for application, excess removed with sterile gauze, reusable ancillary items reprocessed on site) versus a single-use pre-filled applicator (ChloraPrep®, Becton Dickinson) containing the same antiseptic. The antiseptic, its concentration and the standardized back-and-forth technique were identical in both arms, so that the comparison is between the two complete workflows.
**Perspective**	8	State the perspective(s) adopted by the study and why chosen.	Section 2.4. Hospital provider perspective, per procedure, because the resources affected by the delivery format (materials, staff time and operating room occupancy) are borne by the provider.
**Time horizon**	9	State the time horizon for the study and why appropriate.	Section 2.4. Limited to the intraoperative skin-antisepsis workflow, because the comparison concerns the delivery format only and no downstream clinical outcome was measured.
**Discount rate**	10	Report the discount rate(s) and reason chosen.	Section 2.4. No discounting was applied, given the intraoperative, sub-year time horizon.
**Selection of outcomes**	11	Describe what outcomes were used as the measure(s) of benefit(s) and harm(s).	Sections 2.2, 2.3 and 2.4. Operational and economic outcomes: workflow time by step and in total (excluding the air-drying interval); residual alcoholic chlorhexidine, aqueous pre-scrub solution, solid waste and total measured waste; and direct per-procedure cost. No health outcome or utility was measured; the analysis is conditional on the antimicrobial equivalence of application methods reported elsewhere (Section 4.5).
**Measurement of outcomes**	12	Describe how outcomes used to capture benefit(s) and harm(s) were measured.	Sections 2.2 and 2.3; Supplementary Section S1. Times measured prospectively by stopwatch against predefined intervals, identical in both arms; residues weighed on a calibrated electronic balance using the definitions and procedures given in Section S1.
**Valuation of outcomes**	13	Describe the population and methods used to measure and value outcomes.	Not applicable. No health benefit was valued; resource use was valued as described for item 14.
**Measurement and valuation of resources and costs**	14	Describe how costs were valued.	Section 2.4; Tables 1 and 2; Supplementary Section S2. Bottom-up micro-costing of directly observed resource use, following published recommendations for micro-costing studies. Material unit prices from the Hospital Pharmacy price list; personnel and operating room costs per minute from the hospital's Analytical Accounting and Profitability

			Department (fully loaded rates: medical staff €1.63/min, nursing €1.20/min per nurse; personnel rate €8.49/min for two surgeons, one anesthesiologist and three nurses; combined personnel and operating room rate €12.89/min). Reprocessing of reusable items allocated by tray-space share of a €45 sterilization container (Section S2). Quantities and unit costs are reported separately. The tariff structure of a second hospital of the same institution was used in a transferability scenario (Table S5).
**Currency, price date, and conversion**	15	Report the dates of the estimated resource quantities and unit costs, plus the currency and year of conversion.	Section 2.4. Resource quantities measured in April–June 2024; unit costs current at the time of data collection; all costs in 2024 euros; no currency conversion.
**Rationale and description of model**	16	If modelling is used, describe in detail and why used. Report if the model is publicly available and where it can be accessed.	Not applicable. No decision-analytic or simulation model was used; costs were built up directly from observed quantities and unit prices. The deterministic cost calculation is publicly available as a spreadsheet (File S1; Zenodo).
**Analytics and assumptions**	17	Describe any methods for analysing or statistically transforming data, any extrapolation methods, and approaches for validating any model used.	Section 2.5. Differences in means (applicator minus bulk bottle) with BCa and percentile bootstrap 95% confidence intervals (10,000 resamples stratified by arm, seed 2,000,000), exact permutation tests on the mean difference (184,756 allocations) and exact Mann–Whitney U tests; Fisher–Freeman–Halton exact test for procedure type. IBM SPSS Statistics 30; percentile intervals verified independently in Python (SciPy). No extrapolation beyond the observed procedures.
**Characterizing heterogeneity**	18	Describe any methods used for estimating how the results of the study vary for sub-groups.	Sections 2.5 and 3.6; Table 7; Tables S3 and S5. No patient subgroup analyses. Heterogeneity by case mix was examined in a post hoc analysis restricted to unilateral hernia repair (7 vs 4) and in a within-arm comparison by prepared field size; outcomes by procedure type are given descriptively in Table S3. Variation across settings was explored with alternative staffing complements and the tariffs of a second hospital (Table S5).
**Characterizing distributional effects**	19	Describe how impacts are distributed across different individuals or adjustments made to reflect priority populations.	Not applicable. No distributional or equity analysis was undertaken; the comparison concerns a procedural step applied uniformly, and no patient-level characteristics were collected.
**Characterizing uncertainty**	20	Describe methods to characterize any sources of uncertainty in the analysis.	Sections 2.4, 2.5, 3.5 and 3.6; Tables 6 and 7; Tables S4–S7; Figures S1–S3. Sampling uncertainty: BCa and percentile bootstrap intervals and exact permutation tests; the intervals are likely too narrow because operators

			were nested within arms, as stated wherever they are reported. Parameter uncertainty: one-way ±20% analyses (Table S4), staffing and tariff scenarios (Table S5), threshold analyses (Table S6) and a two-way analysis of applicator price and combined time rate (Table S7). Regulatory scenario with a medicinal-grade bulk product (Table 6). Case-mix sensitivity analysis (Table 7).
**Approach to engagement with patients and others affected by the study**	21	Describe any approaches to engage patients or service recipients, the general public, communities, or stakeholders (e.g., clinicians or payers) in the design of the study.	No formal patient or public involvement was undertaken. The measurement procedures and the standardized technique were developed in the pilot phase with the participation of the surgical and nursing staff (Sections 2.1 and 3.1).
**RESULTS**			
**Study parameters**	22	Report all analytic inputs (e.g., values, ranges, references) including uncertainty or distributional assumptions.	Section 2.4; Tables 1 and 2; Tables S4–S7. All unit costs, quantities, ranges and alternative values used in the analyses.
**Summary of main results**	23	Report the mean values for the main categories of costs and outcomes of interest and summarise them in the most appropriate overall measure.	Sections 3.3–3.6; Tables 4–7. Mean workflow time by step and in total, decomposed into steps absent from the applicator workflow and steps common to both (Table 4); residual antiseptic and waste (Table 5); per-procedure cost by component for the base case and the medicinal-grade scenario (Table 6); restricted analysis (Table 7). The overall measure is the incremental cost per procedure with its 95% confidence interval; the material-cost difference is reported as a co-primary result.
**Effect of uncertainty**	24	Describe how uncertainty about analytic judgments, inputs, or projections affect findings. Report the effect of choice of discount rate and time horizon, if applicable.	Sections 3.5 and 3.6; Tables S4–S7; Figures S1–S3. Direction of the result under each parameter variation and break-even values. Workflow time is the decisive parameter, and among comparable procedures the observed time difference lies within the range that would reverse the cost result. Discount rate and time horizon: not applicable.
**Effect of engagement with patients and others affected by the study**	25	Report on any difference patient/service recipient, general public, community, or stakeholder involvement made to the approach or findings of the study.	No patient or public involvement. Involvement of surgical and nursing staff in the pilot phase shaped the timing intervals and the standardized technique (Section 2.1).
**DISCUSSION**			
**Study findings, limitations,**	26	Report key findings, limitations, ethical or equity considerations not captured, and how	Section 4. Main findings (4.1), comparison with previous studies (4.2), waste and environmental implications (4.3), economic interpretation and transferability (4.4) and limitations (4.5), including operator nesting, case

**generalizability, and current knowledge**		these could impact patients, policy, or practice.	mix, sequential observation and observer learning, absence of blinding, the single-center setting and center-specific bulk protocol, institution-specific unit costs, exclusion of drying time, absence of clinical endpoints and the role of the funder. The Conclusions (Section 5) state what a confirmatory study should measure.
**OTHER RELEVANT INFORMATION**			
**Source of funding**	27	Describe how the study was funded and any role of the funder in the identification, design, conduct, and reporting of the analysis.	Funding statement after the Abstract; Funding section; Section 4.5. Funded by Becton Dickinson (grant ECOPACT), manufacturer of the applicator evaluated. The funder defined the study objectives, had no access to the data, did not review the manuscript before submission and had no role in the decision to publish. The article processing charge was covered by discount vouchers awarded to the authors for peer review; the funder did not contribute to it.
**Conflicts of interest**	28	Report authors conflicts of interest according to journal or International Committee of Medical Journal Editors requirements.	Conflicts of Interest statement, according to the requirements of the journal and the ICMJE: F.G.-G. received personal fees from Becton Dickinson for presenting preliminary results at a company-sponsored symposium in 2024; the other authors declare no conflicts of interest.

Table S3. Outcomes by procedure type and delivery system

Procedure	Delivery system	n	Workflow time (s)	Total measured waste (g)	Residual alcoholic chlorhexidine (mL)	Total cost, base case (€)
Unilateral hernia	Applicator	7	108.43	105.00	18.37	30.22
Unilateral hernia	Bulk bottle	4	115.75	149.75	65.06	32.97
Bilateral hernia	Bulk bottle	3	135.67	126.67	45.46	37.25
Incisional hernia	Bulk bottle	1	125.00	177.00	87.50	34.95
HIPEC	Bulk bottle	1	186.00	178.00	93.18	48.06
Gastric bypass	Applicator	1	127.00	114.00	14.64	34.21
Gastric bypass	Bulk bottle	1	253.00	180.00	86.36	62.45
Gall bladder	Applicator	1	103.00	102.00	16.91	29.06
Pancreas	Applicator	1	147.00	140.00	20.32	38.51

Values are means. Cells of one to seven procedures are presented for transparency and do not support inference. HIPEC = hyperthermic intraperitoneal chemotherapy (Sugarbaker procedure). The longest single observation in the study was the bulk-bottle gastric bypass (253 s), 110 s above the bulk-bottle mean; the corresponding applicator procedure took 127 s. Neither procedure is included in the restricted analysis (manuscript Section 3.6).

Table S4. One-way sensitivity analysis: effect of ±20% variation in each cost parameter on the incremental cost per procedure (applicator − bulk bottle), €2024.

Parameter varied	Base-case value	Incremental cost at −20% (€)	Incremental cost at +20% (€)	Range (€)
**(a) Bulk-bottle arm using biocide-registered chlorhexidine — base case: −7.57 €**				
Personnel wage rate	€8.49/min	−6.73	−8.42	1.69
Operating room cost per minute	€4.40/min	−7.13	−8.01	0.87
Material unit prices	€8.10 vs €6.93/procedure	−7.34	−7.81	0.47
**(b) Bulk-bottle arm using medicinal-grade chlorhexidine — base case: −9.66 €**				
Personnel wage rate	€8.49/min	−8.82	−10.51	1.69
Material unit prices	€10.19 vs €6.93/procedure	−9.01	−10.31	1.30
Operating room cost per minute	€4.40/min	−9.22	−10.10	0.87

Each parameter was varied independently while all others were held at their base-case values. Negative values denote a lower cost per procedure with the applicator. Parameters are listed in descending order of influence. Material unit prices were varied jointly in both arms. Varying the reprocessing allocation alone (€5.41 per procedure; Section S2) by ±20% gives −6.49 and −8.65 (range 2.16) in panel (a) and −8.58 and −10.74 (range 2.16) in panel (b), without reversing the direction of the result; at −20% the material difference falls to −€0.09 (Table S6, panel c).

Table S5. Scenario analysis: effect of alternative staffing complements and institutional tariffs on the incremental cost per procedure (applicator − bulk bottle), €2024.

Scenario	Medical rate (€/min)	Nursing rate (€/min)	Personnel rate (€/min)	OR	Combined rate (€/min)	Incremental cost, biocide registered bulk (€)	Incremental cost, medicinal grade bulk (€)
**(a) Study site (Pamplona)**							
Base case: senior medical staff	1.63	1.20	8.49	4.40	12.89	−7.57	−9.66
Junior medical staff	1.03	1.20	6.69	4.40	11.09	−6.68	−8.77
Nursing staff only	—	1.20	3.60	4.40	8.00	−5.14	−7.23
**(b) Second site (Madrid)**							
Senior medical staff	1.93	1.60	8.99	6.40	15.39	−8.81	−10.90
Junior medical staff	1.36	1.60	7.28	6.40	13.68	−7.96	−10.05
Nursing staff only	—	1.60	3.20	6.40	9.60	−5.94	−8.03
**(c) Common scenario**							
Time assigned no opportunity value	—	0.00	0.00	0.00	0.00	−1.17	−3.26

Scenarios differ only in the unit costs applied to personnel and to operating room occupancy; the observed workflow times and material costs are identical throughout. Panel (a) varies the personnel complements valued during skin antisepsis while holding the study-site tariffs constant. Panel (b) re-costs the same observed procedures using the complete tariff structure of a second hospital of the same institution, located in a different Spanish region and subject to a different collective agreement; no procedures were observed at that site. The same corporate analytical accounting department produces unit costs for both sites using an identical imputation methodology, making the two tariff structures directly comparable. The personnel rate is calculated as 3 × the medical rate (2 surgeons and 1 anesthesiologist) plus the nursing rate × the number of nurses forming the standard operating room allocation, which is 3 at the study site (1 scrub, 1 circulating and 1 anesthesia nurse) and 2 at the second site, where the anesthesia nurse is not part of that allocation. The combined rate is the personnel rate plus the operating room rate, which covers installations and equipment. In the nursing-only scenario, the combined rate therefore represents the cost of the operating room minute alone, that is, operating room nursing together with installations and equipment, with no medical time counted. Panel (c) sets the opportunity value of both personnel and operating room time to zero, so that only the material difference remains; this scenario is identical at both sites and isolates the component of the difference that corresponds to avoidable expenditure. Negative values denote a lower cost per procedure with the applicator. Scenario estimates are deterministic recalculations from the observed mean times; confidence intervals are reported only for the base case (Table 6), since the scenarios substitute alternative unit costs rather than resampling observed data.

Table S6. Threshold (break-even) analysis: value each parameter would have to reach for the incremental cost (panels a and b) or the material cost difference (panel c) to fall to zero, all other parameters held at base-case values.

Parameter varied	Base-case value	Break-even value	Change from base case
**(a) Bulk arm: biocide-registered chlorhexidine (base case −€7.57)**			
Applicator unit price	€6.93	€14.50	+109.3%
Bulk-bottle material cost	€8.10	€0.53	−93.5%
Workflow time difference	−29.8 s (applicator faster)	+5.4 s (applicator slower)	35.2 s
Combined OR + personnel rate	€12.89/min	Not attainable	—
Reprocessing allocation (bulk arm)	€5.41	Not attainable*	—
**(b) Bulk arm: medicinal-grade chlorhexidine (base case −€9.66)**			
Applicator unit price	€6.93	€16.59	+139.4%
Bulk-bottle material cost	€10.19	€0.53	−94.8%
Workflow time difference	−29.8 s (applicator faster)	+15.2 s (applicator slower)	45.0 s
Combined OR + personnel rate	€12.89/min	Not attainable	—
Reprocessing allocation (bulk arm)	€5.41	Not attainable*	—
**(c) Material cost difference only (base case −€1.17; medicinal-grade scenario −€3.26)**			
Reprocessing allocation, biocide-registered bulk	€5.41	€4.24	−21.6%
Reprocessing allocation, medicinal-grade bulk	€5.41	€2.15	−60.3%
Applicator unit price, biocide-registered bulk	€6.93	€8.10	+16.9%
Applicator unit price, medicinal-grade bulk	€6.93	€10.19	+47.0%

* Reprocessing allocation: the per-procedure share of the sterilization-container cost allocated to the washcloth, gallipot and gripper (€5.41; Section S2). Even at a zero reprocessing cost the incremental total cost remains negative (−€2.16 in the base case; −€4.25 under the medicinal-grade scenario), so no break-even value is attainable for total cost. The material cost difference, by contrast, reaches zero at the values shown in panel (c) and reverses below them, so that with a zero marginal reprocessing cost the applicator is €4.24 (€2.15 under the medicinal-grade scenario) more expensive in materials. OR = operating room.

Table S7. Two-way sensitivity analysis: incremental cost per procedure (applicator − bulk bottle, €2024) across combinations of the combined operating room + personnel rate and the applicator unit price. Negative values favor the applicator.

	Applicator unit price (€)

Combined rate (€/min)	€5.50	€6.93 (base)	€9.00	€12.00	€15.00
**(a) Bulk arm: biocide-registered (€8.10)**					
€0.00 (time has no value)	−2.60	−1.17	+0.90	+3.90	+6.90
€6.00	−5.58	−4.15	−2.08	+0.92	+3.92
€12.89 (base case)	−9.00	−7.57	−5.50	−2.50	+0.50
€15.39 (second site)	−10.24	−8.81	−6.74	−3.74	−0.74
€18.00	−11.54	−10.11	−8.04	−5.04	−2.04
€24.00	−14.52	−13.09	−11.02	−8.02	−5.02
**(b) Bulk arm: medicinal-grade (€10.19)**					
€0.00 (time has no value)	−4.69	−3.26	−1.19	+1.81	+4.81
€6.00	−7.67	−6.24	−4.17	−1.17	+1.83
€12.89 (base case)	−11.09	−9.66	−7.59	−4.59	−1.59
€15.39 (second site)	−12.33	−10.90	−8.83	−5.83	−2.83
€18.00	−13.63	−12.20	−10.13	−7.13	−4.13
€24.00	−16.61	−15.18	−13.11	−10.11	−7.11

Each cell shows the incremental cost per procedure (applicator − bulk bottle) for the corresponding combination of the combined operating room plus personnel rate (rows) and the applicator unit price (columns). Negative values denote a lower cost per procedure with the applicator. All other parameters were held at their base-case values.

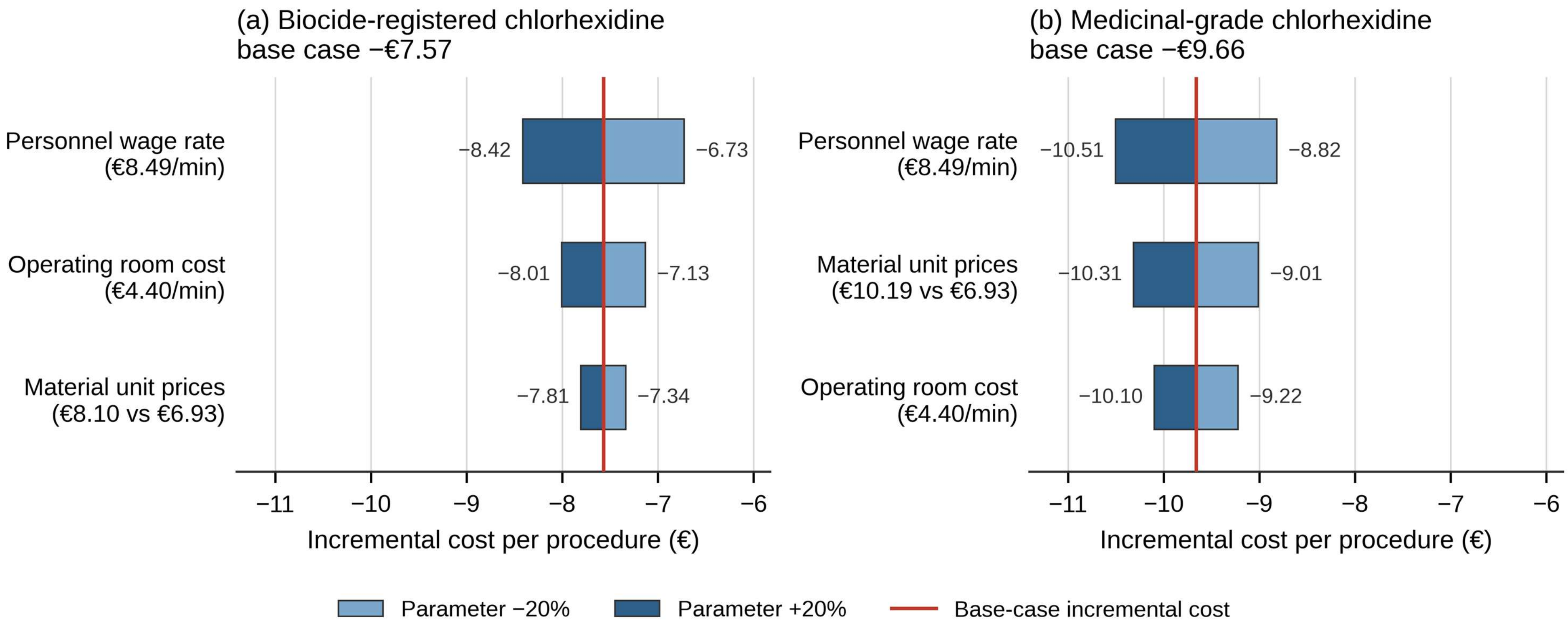

Figure S1. One-way sensitivity analysis of the incremental cost per procedure (applicator − bulk bottle), €2024. Each bar shows the effect of varying a single parameter by ±20% while holding all others at base-case values. The vertical red line marks the base-case incremental cost. (a) Bulk-bottle arm using biocide-registered chlorhexidine; (b) bulk-bottle arm using medicinal-grade chlorhexidine

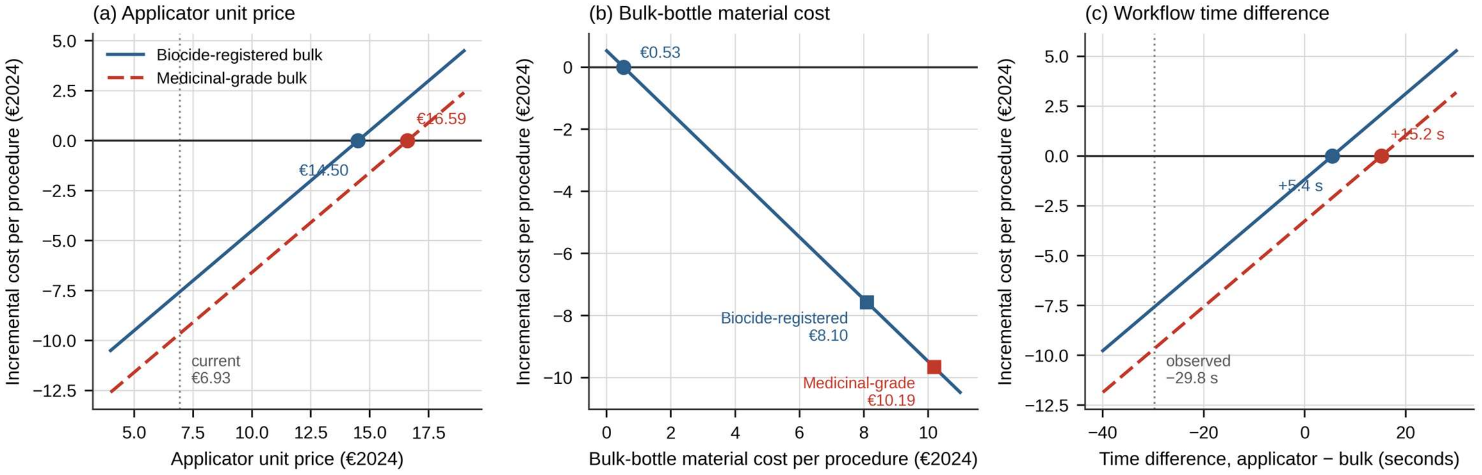

Figure S2. Threshold analysis of the incremental cost per procedure (applicator − bulk bottle), €2024. Each panel varies one parameter across a plausible range while holding all others at base-case values; the horizontal line at zero marks break-even and circles mark the break-even value. Dotted vertical lines and squares indicate observed base-case values. (a) Applicator unit price; (b) bulk-bottle material cost per procedure; (c) workflow time difference between systems.

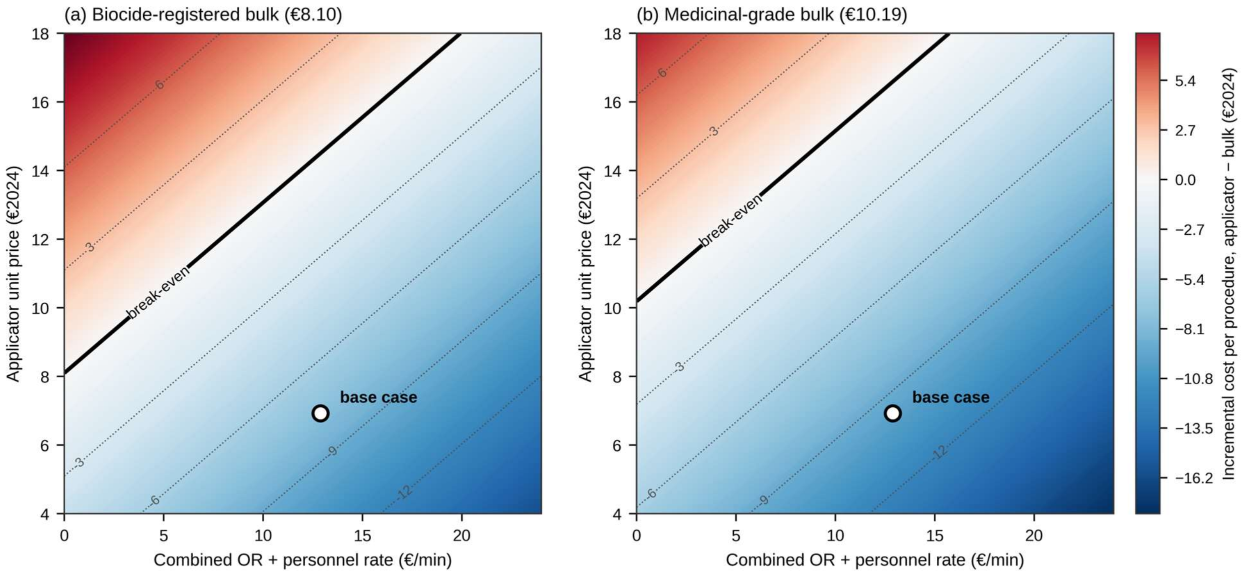

Figure S3. Two-way sensitivity analysis of the incremental cost per procedure (applicator − bulk bottle), €2024, across the combined operating room plus personnel rate (x-axis) and the applicator unit price (y-axis). Blue shading denotes a lower cost per procedure with the applicator and red shading a higher cost; the solid black line is the break-even contour (incremental cost = €0), and the dotted lines are additional iso-cost contours labeled in euros. The white circle marks the observed base-case combination (€12.89 per minute; €6.93 per applicator). (a) Bulk-bottle arm costed with biocide-registered chlorhexidine (€8.10 per procedure); (b) bulk-bottle arm costed with medicinal-grade chlorhexidine (€10.19 per procedure).
